# *Moniliophthora perniciosa*, the Causal Agent of Cacao *Witches’ Broom Disease* Is Killed *in vitro* by *Saccharomyces cerevisiae* and *Wickerhamomyces anomalus* Yeasts

**DOI:** 10.3389/fmicb.2021.706675

**Published:** 2021-09-22

**Authors:** Pedro Ferraz, Rogelio Lopes Brandão, Fernanda Cássio, Cândida Lucas

**Affiliations:** ^1^Institute of Science and Innovation for Bio-Sustainability (IB-S), University of Minho-Campus de Gualtar, Braga, Portugal; ^2^Centre of Molecular and Environmental Biology (CBMA), Department of Biology, University of Minho-Campus de Gualtar, Braga, Portugal; ^3^Nucleus of Research in Biological Sciences, Federal University of Ouro Preto, Ouro Preto, Brazil

**Keywords:** antagonism, cacao, *Witches’ Broom Disease*, *Moniliophthora perniciosa*, fermentative yeasts, necrotrophic mycoparasite, *Saccharomyces cerevisiae* PE2, *Wickerhamomyces anomalus* LBCM1105

## Abstract

Cacao plantations from South America have been afflicted with the severe fungal disease known as *Witches’ Broom Disease* (WBD), caused by the basidiomycete *Moniliophthora perniciosa*. Yeasts are increasingly recognized as good fungal biocides, although their application is still mostly restricted to the postharvest control of plant and fruit decay. Their possible utilization in the field, in a preharvest phase, is nevertheless promising, particularly if the strains are locally adapted and evolved and if they belong to species considered safe for man and the environment. In this work, a group of yeast strains originating from sugarcane-based fermentative processes in Brazil, the cacao-producing country where the disease is most severe, were tested for their ability to antagonize *M. perniciosa in vitro*. *Wickerhamomyces anomalus* LBCM1105 and *Saccharomyces cerevisiae* strains LBCM1112 from spontaneous fermentations used to produce *cachaça*, and PE2 widely used in Brazil in the industrial production of bioethanol, efficiently antagonized six strains of *M. perniciosa*, originating from several South American countries. The two fastest growing fungal strains, both originating from Brazil, were further used to assess the mechanisms underlying the yeasts’ antagonism. Yeasts were able to inhibit fungal growth and kill the fungus at three different temperatures, under starvation, at different culture stages, or using an inoculum from old yeast cultures. Moreover, SEM analysis revealed that *W. anomalus* and *S. cerevisiae* PE2 cluster and adhere to the hyphae, push their surface, and fuse to them, ultimately draining the cells. This behavior concurs with that classified as necrotrophic parasitism/mycoparasitism. In particular, *W. anomalus* within the adhered clusters appear to be ligated to each other through roundish groups of fimbriae-like structures filled with bundles of microtubule-sized formations, which appear to close after cells detach, leaving a scar. SEM also revealed the formation of tube-like structures apparently connecting yeast to hypha. This evidence suggests *W. anomalus* cells form a network of yeast cells connecting with each other and with hyphae, supporting a possible cooperative collective killing and feeding strategy. The present results provide an initial step toward the formulation of a new eco-friendly and effective alternative for controlling cacao WBD using live yeast biocides.

## Introduction

The cacao plant (*Theobroma cacao* L.) is one of the most valuable crops worldwide (Pohlan and Pérez, 2010; Teixeira et al., 2015), responsible for the important parts of the economic revenue of countries from Central and South America and Africa within the Cacao Belt. *T. cacao* is affected by several diseases, the most severe being the *Witches’ Broom Disease* (WBD), caused by the basidiomycete fungal phytopathogen *Moniliophthora perniciosa* (formerly *Crinipellis perniciosa*; Purdy and Schmidt, 1996; Aime and Phillips-Mora, 2005; Teixeira et al., 2015). WBD was responsible for major crop losses particularly in Brazil, where the cacao production decreased more than 70% during the 10years that followed the onset of the disease (Trevizan and Marques, 2002; Meinhardt et al., 2008; Teixeira et al., 2015). Large social-economic consequences accompanied this fall, mainly in the region of Bahia, where production losses of around 90% in the first years of the disease onset led to more than 200,000 farmers to lose their job and economic support (Trevizan and Marques, 2002; Teixeira et al., 2015).

The severity of WBD derives from the extreme virulence of *M. perniciosa*, which infects all cacao plant tissues at all stages of the plant life cycle (Meinhardt et al., 2008; Ferraz et al., 2019). As a hemibiotrophic fungus, *M. perniciosa* has two distinct phases: a biotrophic and a saprotrophic (reviewed by Ferraz et al., 2019). After the initial infection, the pathogen induces hypertrophy and hyperplasia, causing a disorganized proliferation of the infected vegetative meristems of axillary shoots, which results in the formation of green brooms, a structure composed of abnormal stems. Several weeks after the development of these structures, the infected plant tissues become necrotic due to a series of cell death events, forming a structure named dry broom (Meinhardt et al., 2008). *M. perniciosa* then colonizes those necrotic plant cells and generates pink-colored basidiocarps producing 2 to 3.5 million spores each (Almeida et al., 1997). The spores are mainly released at night, under optimal conditions of temperature and humidity, and disseminated by water and wind, and can endure and remain latent in the soil or inside pruned plant branches for long periods of time (Meinhardt et al., 2008; Pohlan and Pérez, 2010). All these factors contribute to the exceptional virulence of *M. perniciosa* and explain why a whole plantation is compromised after the initial infection of one cacao plant.

The conventional chemical fungicides used to control the spread of fungal plant diseases, such as copper or azole-based compounds, are ineffective against *M. perniciosa* (Medeiros et al., 2010). In addition, the use of this type of chemicals has been restricted in most cacao-producing countries, due to high cost and risks associated with contamination of the cacao fruit and chocolate (Marelli et al., 2009; Verweij et al., 2009; Nunes, 2012). Currently, the only WBD management strategy implemented in Brazil consists of spraying the infected plants with a suspension of *Trichoderma stromaticum* (Tricovab^®^), which is a fungal competitor of *M. perniciosa* (Ferraz et al., 2019). Its production is very expensive and unpractical, being therefore heavily subsidized by the Brazilian government (reviewed by Ferraz et al., 2019). New and more sustainable strategies to control the disease are essential. One such alternative could be the use of biological control agents (BCA), that is, microbes that antagonize the fungus, contributing in the long run to contain or suppress the development of the phytopathogen (Pal and McSpadden Gardener, 2006; Ferraz et al., 2019; Freimoser et al., 2019).

Many yeasts are biocides and are used as BCA of fungal deterioration of food products in the postharvest phase, decreasing important economic losses during transport and storage (Marquina et al., 2002; Liu et al., 2013; Dukare et al., 2018). Yeast biocides are best known for being Killers, that is, yeasts that antagonize other yeasts through the secretion of peptides that act as Killer toxins (reviewed by Liu et al., 2015). Broad Killer yeasts not only act upon a wide range of other yeasts from genetically distant species, but may also be effective against bacteria and filamentous fungi (Liu et al., 2015) making use of a panoply of other mechanisms (reviewed by Freimoser et al., 2019). Yeasts may compete for space or nutrients, either passively, by growing faster than fungi, or actively, by secreting siderophores that chelate ferric ions which are vital for fungal development (Magliani et al., 1997; Nally et al., 2015; Fialho et al., 2016). Otherwise, they may secrete hydrolytic enzymes, such as chitinases and glucanases (Lopes et al., 2015; Liu et al., 2018), which attack the fungal cell wall. They may also secrete volatile compounds (VOCs), including alkenes, alcohols, ketones, benzenoids, pyrazines, sulfides, and terpenes (Schulz-Bohm et al., 2017), each with a specific deleterious action upon each fungal species (Nally et al., 2015). Often, antagonism is achieved through more than one simultaneous mechanism (Walker, 2011; Pretscher et al., 2018). Additionally, a few yeasts from a same clade, containing a majority of *Saccharomycopsis*, were described as predacious yeasts, killing other yeast cells as mycoparasites, by penetrating them with haustoria/penetration pegs (Lachance and Pang, 1997; Lachance et al., 2000). Conceptually, mycoparasitism follows 5 stages: (i) mutual recognition of parasite and prey, (ii), physical contact between the two, (iii) secretion of compounds and/or proteins directed to destroy the fungal cell wall, (iv) penetration of the fungal cell, and (v) cell draining/lysis/death of the fungus (Dukare et al., 2018). There are several types of mycoparasites, including the necrotrophic which are very aggressive organisms that may act upon a broad range of fungal preys. They can attack filamentous fungi at a distance by secreting toxins or lytic enzymes into the surrounding environment (Junker et al., 2019), or they can act by direct physical contact, although they do not necessarily invade the target cell (Mims et al., 2007). The ones that do are known as invasive necrotrophs or predatory mycoparasites. They penetrate their prey fungal cells through haustoria or penetration pegs (Junker et al., 2019), killing the fungus or feeding from its cells, or both (Jeffries, 1995; Junker et al., 2019). Although in literature yeasts are often termed as mycoparasites of filamentous fungi, it is not clear whether they follow these stages toward their targets, whether they ever penetrate the preyed fungal cells, or whether they feed on it as a true predator.

Dominance processes of this kind must play important roles in microbial ecology, not only in natural niches but also in spontaneous fermentations (Abranches et al., 1998), in which prevailing fermentative strains commonly antagonize other yeasts and bacteria (Alonso-del-Real et al., 2019). Many industrial strains originate from such environments (Lopes et al., 2016), providing a valuable source for microbial biodiversity with interesting and useful natural properties for many applications (da Conceição et al., 2015), including the biocontrol of fungal infections in animals and plants (Hatoum et al., 2012; Mannazzu et al., 2019). In the particular case of WBD, *M. perniciosa* was previously reported to be inhibited *in vitro* by two yeast isolates from *Candida* sp. and *Dipodascus capitatus* (Cabral et al., 2009), although their ability was not explored in detail, and their mode of action remains unknown. In this study, we hypothesized that yeasts might be able to antagonize *M. perniciosa.* For that, yeast isolates from sugarcane-based fermentative processes were used to test antagonism against *M. perniciosa*. Three strains, one of *Wickerhamomyces anomalus* and two of *Saccharomyces cerevisiae,* were found to kill this phytopathogen in an efficient and resilient manner. In particular, *W. anomalus* displayed toward the hyphae a behavior characteristic of a predacious, necrotrophic mycoparasite. Their biocidal potential raises expectations as to their possible application in the management of this severe cacao disease, until now without effective or sustainable methods for its containment.

## Materials and Methods

### Microorganisms and Culture Conditions

The *M. perniciosa* strains originally isolated from cacao plants, and fruits infected with WBD in South American countries (Table 1) were purchased from KNAW.^1^ They were cryopreserved in sterile glycerol 30% at −80°C, maintained at 4°C on MEA (20g/l malt extract with 20g/l agar), and propagated in the same media or in PDA at 30°C. Alternatively, filamentous fungi were grown in liquid ME (20g/l malt extract), using glass tubes (13cm×Ø 3cm) containing 20ml of medium at the same temperature and 200rpm orbital shaking. Media pH was adjusted to the desired value with NaOH 2M or HCl 37% v/v. Both solid and liquid media were inoculated using a≈0.8×0.8cm MEA plug with actively growing mycelia (not older than 1week). Growth on solid media was followed measuring the mycelium diameter (G_d_) every 24h under a Stereo Zoom Binocular Microscope (Leica s8 APO), and growth rate (G_r_) was estimated as the ratio G_d_ (mm)/t (day). Latency phase was not considered for this calculation. Growth in liquid media was visually inspected, mycelia forming one or more cotton ball-like conglomerates.

**Table 1:** Strains of *Moniliophthora perniciosa* from CBS-KNAW (www.wi.knaw.nl).

Strain	Country of origin
CBS 441.80	Brazil
CBS 442.80	Brazil
CBS 192.77	Ecuador
CBS 193.77	Ecuador
CBS 245.36	Ecuador
CBS 789.86	Ecuador
CBS 790.86	Ecuador
CBS 339.50	Venezuela

Yeasts strains originating from diverse sources (Table 2) were cryopreserved in sterile glycerol 30% at −80°C, maintained at 4°C on YPDA (10g/l yeast extract, 10g/l bacto peptone, 20g/l D-glucose with 20g/l agar), and multiplied at 30°C in the same medium for 48–72h prior to assays. Growth in YPD or ME was done at the same temperature with 200rpm orbital shaking and a liquid/air ratio of 1:2.5 and followed spectrophotometrically at 600nm or counting the cells in suspension with a Neubauer Chamber under a Light Microscope (Leica DM 300). Growth rate [μ_g_ (h^−1^)] was calculated from ODt_x_=ODt_0_.e^μg.tx^.

**Table 2:** Yeast strains used in this work, their collection code, their primitive origin, and their assigned code in this work.

Species	Strain	Origin	Code
*Meyerozyma guilliermondii*	LBCM 1015	Fermentations underlying the production of *cachaça*	#1015
*Wickerhamomyces anomalus*	LBCM 1105		#1105
*Saccharomyces cerevisiae*	LBCM 1025		#1025
*Saccharomyces cerevisiae*	LBCM 1038		#1038
*Saccharomyces cerevisiae*	LBCM 1096		#1096
*Saccharomyces cerevisiae*	LBCM 1112		#1112
*Saccharomyces cerevisiae*	LBCM 1113		#1113
*Saccharomyces cerevisiae*	CAT-1- FT280L	Industrial production of bioethanol	CAT1
*Saccharomyces cerevisiae*	PE-2- FT134L		PE2

*Two companies supplied the yeast strains: Cerlev, Lda., Ouro Preto, MG, Brazil* (https://www.facebook.com/empresa.cerlev/) *the upper group of strains coded as LBCM, and Fermentec, Lda., Piracicaba, SP, Brazil* (https://www.fermentec.com.br/capa.asp?pi=principal) *the two remaining strains*.

### Evaluation of the Yeast vs. Filamentous Fungus Antagonistic Ability

#### Solid Media Assays

Antagonism between yeasts and filamentous fungi was assayed in MEA or PDA, at 30°C. A filamentous fungus inoculum plug, as described above, was placed on top of a plate of medium supplemented with 0.015% methylene blue (MB; adapted from Lima et al., 2013). After an initial filamentous fungal growth (~3mm Ø), a yeast strain was inoculated on one side of the plate, corresponding to a generous strikeout of 48h YPD plate cultures. Plates were photographed, and mycelial growth was registered according to an empirical classification scale with 3 levels: Level 0 represents the absence of inhibition of any kind, the filamentous fungus eventually growing on top of the yeast culture; level 1 represents a weak inhibitory response in which case the filamentous fungus grows up to the limit of the yeast culture without overgrowing it; and level 2 represents a clear antagonistic effect.

#### Liquid Media Assays

Antagonism in liquid media was evaluated inoculating an actively growing mycelium plug together with a suspension of ME-grown yeast cultures collected in exponential phase (A_600_ 1.0) to a final concentration of 5×10^6^ cells/ml, using glass tubes (13cm×Ø 3cm) containing 20ml of ME medium, and incubating at 30°C and 200rpm orbital shaking for 10days. As expected, the yeasts grew faster than the fungi, filling the growth medium. After 10days of co-culture, the medium was decanted to check for the presence or absence of mycelium. Tubes were photographed, and growth of mycelia was inspected visually and registered according to an empirical classification scale equivalent to the one used for solid medium: Level 0 represents the absence of inhibition, the fungus growing three dimensionally to form a large conglomerate of hyphae; level 1 corresponds to a weak inhibitory response, with the development of some mycelia around the agar plug; and level 2 represents a strong inhibition with the total absence of mycelial growth. The plug was washed with ultrapure water, softly shaking manually. The procedure was repeated ±10 times to obtain maximum removal of the yeasts attached to the mycelium. The plug was then placed in fresh ME and photographed. For the utilization of supernatants from yeast cultures or yeast/ filamentous fungus co-cultures to test fungal growth inhibition, these were supplemented with ME 2% (w/v) to avoid the starvation of the filamentous fungi during the 10days of assay.

### Assessment of Filamentous Fungal Death by Staining With Methylene Blue and Propidium Iodide

At the end of the incubation period, the viability/death status of the remaining fungal cells was evaluated by staining with MB and propidium iodide (PI). For MB staining, a small portion of the remaining mycelia was collected, washed with ultrapure water, added a drop of MB 0.03% v/v, incubated 10min at room temperature, and observed under a light microscope (Olympus BX63F2 equipped with an Olympus DP74 camera). For PI staining, the filamentous fungal sample was washed with ultrapure water, placed in a microtube containing 500μl PBS (phosphate-buffered saline) and 1μl of PI (1mg/ml), and incubated for 10min in the dark at room temperature. Fluorescence was assessed with an epifluorescence microscope (Olympus BX63F2 equipped with an Olympus DP74 camera), using monochromatic light at 543nm and an emission bandpass filter of 585–615nm.

### SEM Analysis of the Interaction Between Yeast and *M. perniciosa* Cells

The yeasts strains *W. anomalus* #1105 and *S. cerevisiae* PE-2-FT134L (PE2) were used against *M. perniciosa* strains CBS 441.80 and 442.80 in liquid cultures for 10days. Samples were gently washed with ultrapure water and soft manual shaking, fixed in 1ml of 2.5% v/v glutaraldehyde in PBS for 48h at 4°C, rinsed with 1ml distilled water, and postfixed with 1ml of 1% v/v of osmium tetroxide for 1h at room temperature (adapted from das Murtey and Ramasamy, 2016). Samples were subsequently dehydrated through immersion for 20min in a series of ethanol-water solutions (1ml of 20, 30, 40, 55, 70, 80, 90, 95, and 100% v/v of ethanol). Fungal samples were then dried at room temperature and coated with a thin Au/Pd layer using a High Resolution Sputter Coater, 208HR Cressington Company, coupled to a MTM-20 Cressington High Resolution Thickness Controller. Scanning electron microscopic (SEM) assessment was done in a NanoSEM (FEI Nova 200) at a 5 or 10kv voltage with a through-lens detector.

### Statistical Analysis

All assays, including the SEM, were performed at least in three independent replicates (*n*≥3). The data obtained were subjected to a one-way ANOVA using GraphPad Prism 6 (GraphPad Software, Inc.). Statistical significance was assumed at *p*≤0.05.

## Results

### What Are the Optimal Conditions for *in vitro* Cultivation of *M. perniciosa*?

*M. perniciosa* is a very resilient fungus (Purdy and Schmidt, 1996; ICCO.org, 2021), which grows preferably within a relatively narrow range of temperatures (20–30°C). Considering that most yeasts are best cultured at 30°C, this temperature was chosen to cultivate the *M. perniciosa* strains. Optimal pH, on the other hand, was determined by quantifying filamentous fungal growth rates in MEA adjusted to pH 4.0 to 6.0 as in Supplementary Figure 1. In these conditions, *M. perniciosa* fastest grower was the CBS 441.80 strain from Brazil with a specific growth rate of 2.7mmday^−1^ at pH 5, while the strain CBS 193.77 from Ecuador was the slowest, growing at 0.47mmday^−1^ at pH 4.5. Growth rates were generally lowest at pH 4.0, with a latency phase of 1day (CBS 245.36, 789.86 and 441.80) or more (remaining strains). For strains CBS 245.36, 790.86, and 339.50, this latency period was also observed at pH 4.5. These assays were repeated in PDA, and no statistically significant differences were observed between the growth rates in either medium at each pH (not shown). *M. perniciosa* strains were, hence, cultivated on MEA or PDA at 30°C and pH 5.5. From the same assays, it was also established that 10-day incubation is enough under these conditions to test mycelium growth phenotypes.

### Are There Yeasts Able to Antagonize *M. perniciosa in vitro*?

**Figure 1:**
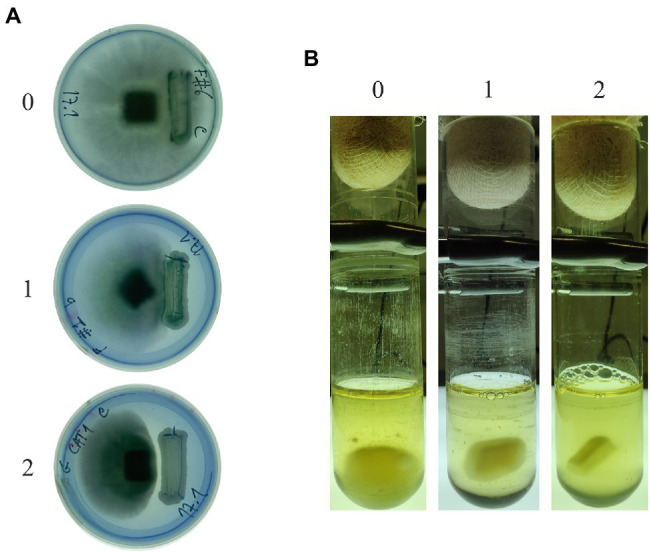
Empirical classification scales of antagonistic response in solid **(A)** and liquid **(B)** ME at 30°C. Level 0 corresponds to a high filamentous fungal resistance (the absence of inhibition); level 1 corresponds to a weak inhibitory effect; and level 2 corresponds to a clear antagonistic growth inhibition effect. In (B), the culture media was replaced with fresh ME to allow visualization of the results without the turbidity resulting from yeast growth.

Yeast strains in Table 2 are isolates from biotechnology companies in Brazil and were chosen based on their dominant nature in microbial mixtures fermenting sugarcane juice (da Conceição et al., 2015; Lopes et al., 2016). Yeasts were firstly challenged with *M. perniciosa* Brazilian strains CBS 441.80 and 442.80 in MEA and PDA, supplemented or not with MB. All, except one, affected the growth of *M. perniciosa* to a different extent. The mycelia developed freely in the opposite direction of the yeast strikeout, while the extent of its development in the space between the yeast and the filamentous fungal plug varied considerably. Based on this variation, three levels of response were identified, which were converted into an empirical scale of yeast/filamentous fungus interaction with three levels (0, 1, and 2) as displayed in Figure 1A. Results obtained in MEA scored in this way are presented in Table 3. Identical results were obtained in PDA and in media without MB (not shown).

**Table 3:** Results of the antagonism assays between *M. perniciosa* and yeasts in solid and liquid media.

Yeast strains	*M. guilliermondii*	*W. anomalus*	*S. cerevisiae*						
	#1015	#1105	#1038	#1096	#1025	#1112	#1113	CAT1	PE2
*M. perniciosa*	Solid medium assays								
CBS 441.80	1	0	1	0	0	0	0	0	0
CBS 442.80	0	0	1/1/2	1	1	0	1	2	1
	Liquid medium assays								
CBS 441.80	2	2	1	0	0	2	0	1	2
CBS 442.80	2	2	1	0	0	2	0	2	2

*The inhibitory effect was rated from 0 to 2, according to the empirical scales in Figure 1. Results presented were identical in three independent replicates. One result displayed as 1/1/2 represents a combination where each plate had a different result, identically occurring in each of the triplicates*.

Since the yeasts are faster growers than fungi, the 10-day incubation needed for fungal mycelium development is unfavorable to the maintenance of a fully viable yeast culture in solid media., and a blue halo of cell death around the yeast biomass may appear (not shown). In some cases, the whole yeast biomass can eventually become blue. For this reason, the possibility of assaying antagonism more efficiently in liquid medium, which provides a direct contact between hyphae and yeasts, was considered. Liquid cultures were tested in the same conditions as solid but with orbital shaking, proving ideal for testing the formation of a fungal biomass of considerable size as exemplified in Figure 1B (left tube). Identically to the plate assays, antagonism in liquid cultures yielded more or less mycelium, and a second 0, 1, and 2 empirical scale was generated to rank antagonism (Figure 1B). Results were scored according to this scale (Table 3), which shows a general increase in the yeasts inhibitory ability, particularly in the combinations using *M. guilliermondii* #1015, *W. anomalus* #1105, and *S. cerevisiae* #1112 and PE2.

In view of these results, the *W. anomalus* #1105 and *S. cerevisiae* #1112 and PE2 were further tested against all the remaining *M. perniciosa* strains in Table 1. Identical maximum inhibition of the growth of all the fungal strains was observed with either yeast strain. This evidence the broadness of these yeasts’ antagonistic ability and confirming the appropriateness of these yeasts for biocidal action against WBD, which is often caused by a mixture of several fungal strains.

### Is Antagonism Maintained in Non-optimal Conditions?

To make a preliminary assessment of the resilience of the antagonistic effect, combinations between yeasts, that showed a strong antagonistic ability and the two *M. perniciosa* strains used above, were challenged with various environmental stressors. The standard assay was done on ME pH 5.5 at 30°C for 10days, and tubes were inoculated with filamentous fungi and fresh exponentially growing yeast. For each of the stressor’s assays, one of the standard conditions was modified: (i) The temperature was lowered to 16°C or 25°C; or the fresh yeasts’ inoculum was replaced by (ii) starved cells (3days in sterile water), (iii) a 10-day-old inoculum from solid medium, or (iv) a stationary phase (3-day-old) liquid medium inoculum. Results are summarized in Table 4. *W. anomalus* #1105 and *S. cerevisiae* #1112 and PE2 maintained their antagonistic ability against both *M. perniciosa* strains in all these non-optimal conditions, while the remaining yeasts (*M. guilliermondii* #1015 and *S. cerevisiae* #1112) did not. Interestingly, the lower temperatures had no influence on the normal growth of both filamentous fungal strains which is a good prognosis for *in field* application.

**Table 4:** Antagonism assays using selected combinations of yeast and filamentous fungal strains, challenged with environmental stressors.

Fungal strains	*M. perniciosa* CBS 441.80 vs					*M. perniciosa* CBS 442.80 vs				
Yeast strains	*M. guilliermondii*	*W. anomalus*	*S. cerevisiae*			*M. guilliermondii*	*W. anomalus*	*S. cerevisiae*		
	#1015	#1105	#1112	CAT1	PE2	#1015	#1105	#1112	CAT1	PE2
Standard assay	2	2	2	1	2	2	2	2	2	2
16°C	2	2	2	0	2	0	2	2	0	2
25°C	2	2	2	0	2	0	2	2	0	2
Starved inoculum	2	2	2	1	2	2	2	2	2	2
Aged inoculum	2	2	2	1	2	2	2	2	1	2
Stationary phase inoculum	2	2	2	0	2	2	2	2	1	2

*Temperatures of 16°C or 25°C; yeasts starved for 3days in sterile water or aged for 10 days in solid medium at room temperature; or yeast inoculum from liquid medium from 3-day-old stationary phase culture. The inhibitory effect was rated according to Figure 1. Identical results were obtained in three independent replicates*.

### Are Yeasts Secreting to the Extracellular Medium Compounds That Inhibit *M. perniciosa*?

**Figure 2:**
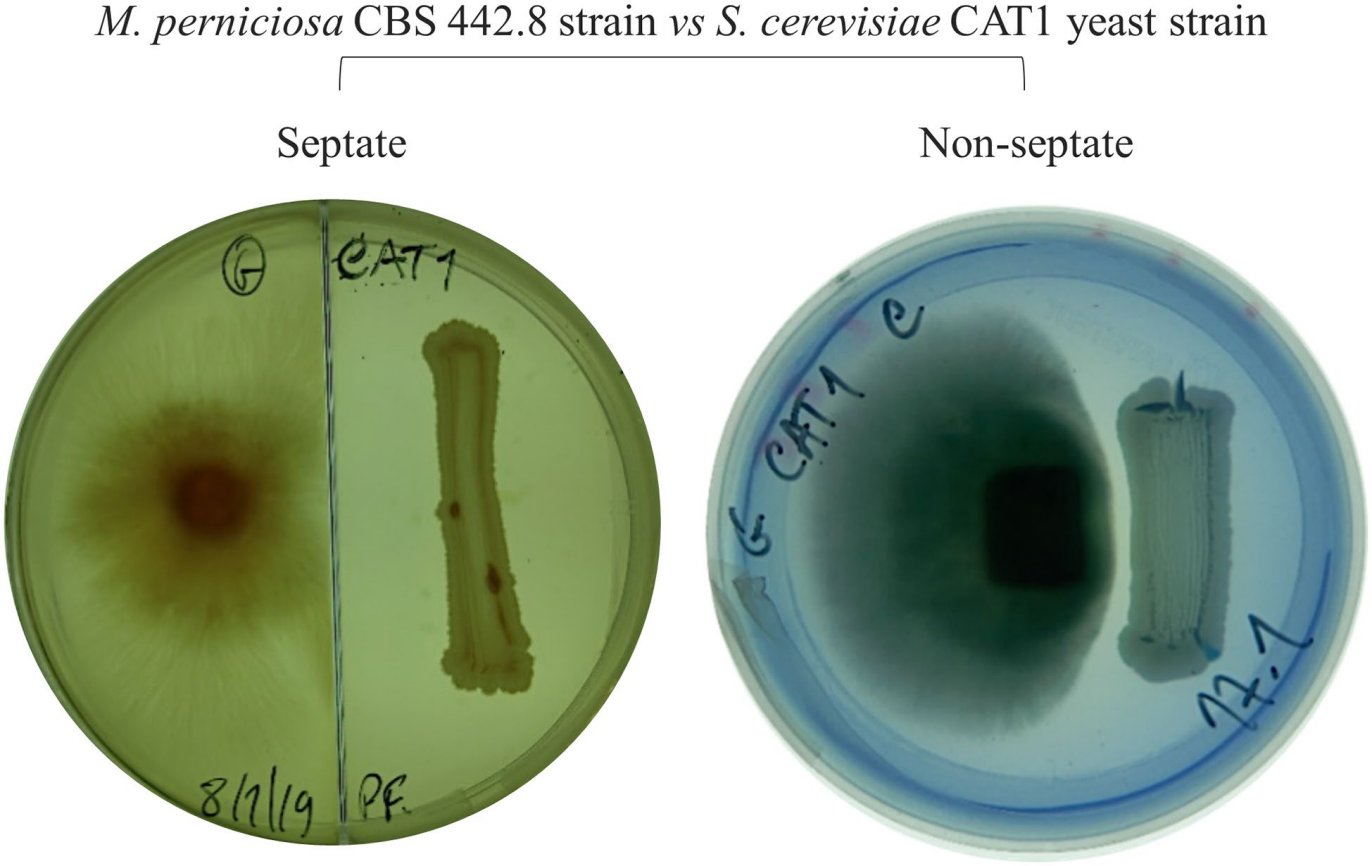
Example of an antagonism assay in septate (left) and in regular (right) MEA plate incubated at 30°C for 7days. The inhibition of *Moniliophthora perniciosa* is not observed in the septate plate.

In plate assays, strong inhibition of mycelium growth occurred at a certain distance between the yeast and the fungal inoculum (e.g., Figure 1A, antagonism level 2). This is compatible with the yeast strains secreting a compound that signals the fungal cells preventing their development in that direction. To evaluate these possibilities, antagonism assays were repeated using septate Petri dishes. These prevent the diffusion of molecules through the agar but allow the organisms in the two sides of the plate to share the atmosphere inside the plate. No inhibition of mycelium development was observed for any of the combinations tested (e.g., Figure 2), indicating that *M. perniciosa* is likely inhibited by a compound that diffuses through the agar.

In what regards liquid medium, although it appears that physical contact between yeast and hypha was required, the possibility that yeasts were secreting to the medium enzymes or compounds that act upon the fungus or as auxiliary of the antagonistic action was assessed. To test this possibility, the supernatants of liquid media yeast cultures or yeast-fungus co-cultures were used to incubate the filamentous fungi as previously. From all the 9 yeast strains and 18 combinations in Table 3, only in one combination fungal growth was inhibited (level 2), that of the supernatant of the co-culture of *M. perniciosa* CBS 442.80 with yeast *S. cerevisiae* #1112, while this did not happen when using the supernatant from the yeast culture alone. This indicates that this yeast secretes enzymes and/or inhibitory compounds, but that the yeast needs the stimulus of the presence of the mycelium to trigger that response. Although it cannot be discarded that the other yeasts also produce identical compounds or enzymes but in amounts that are not enough to preclude mycelium development, results suggest that *S. cerevisiae* #1112 mode of action against *M. perniciosa* is different from the other yeasts.

### Are *M. perniciosa* Hyphae Dying Under the Antagonism Effect of Yeasts?

**Figure 3:**
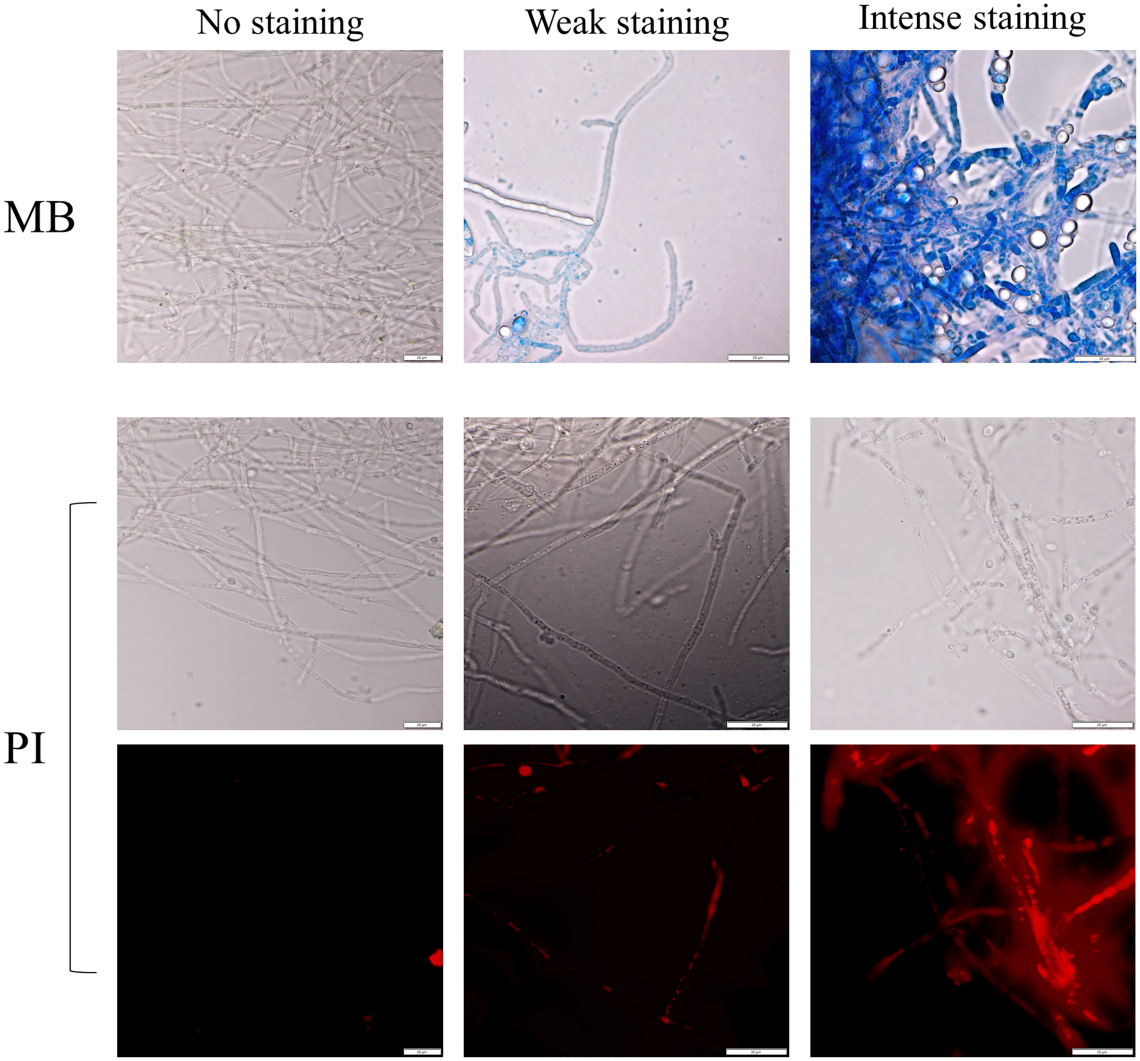
Examples of the yeast/filamentous fungal strains cultures stained with MB (upper panel) and PI [corresponding to the observed bright field (middle panel) and fluorescence (lower panel) of the same picture, respectively]. The absence of staining is compared with two degrees of staining, weak, and intense. Scale bar: 20μm.

**Figure 4:**
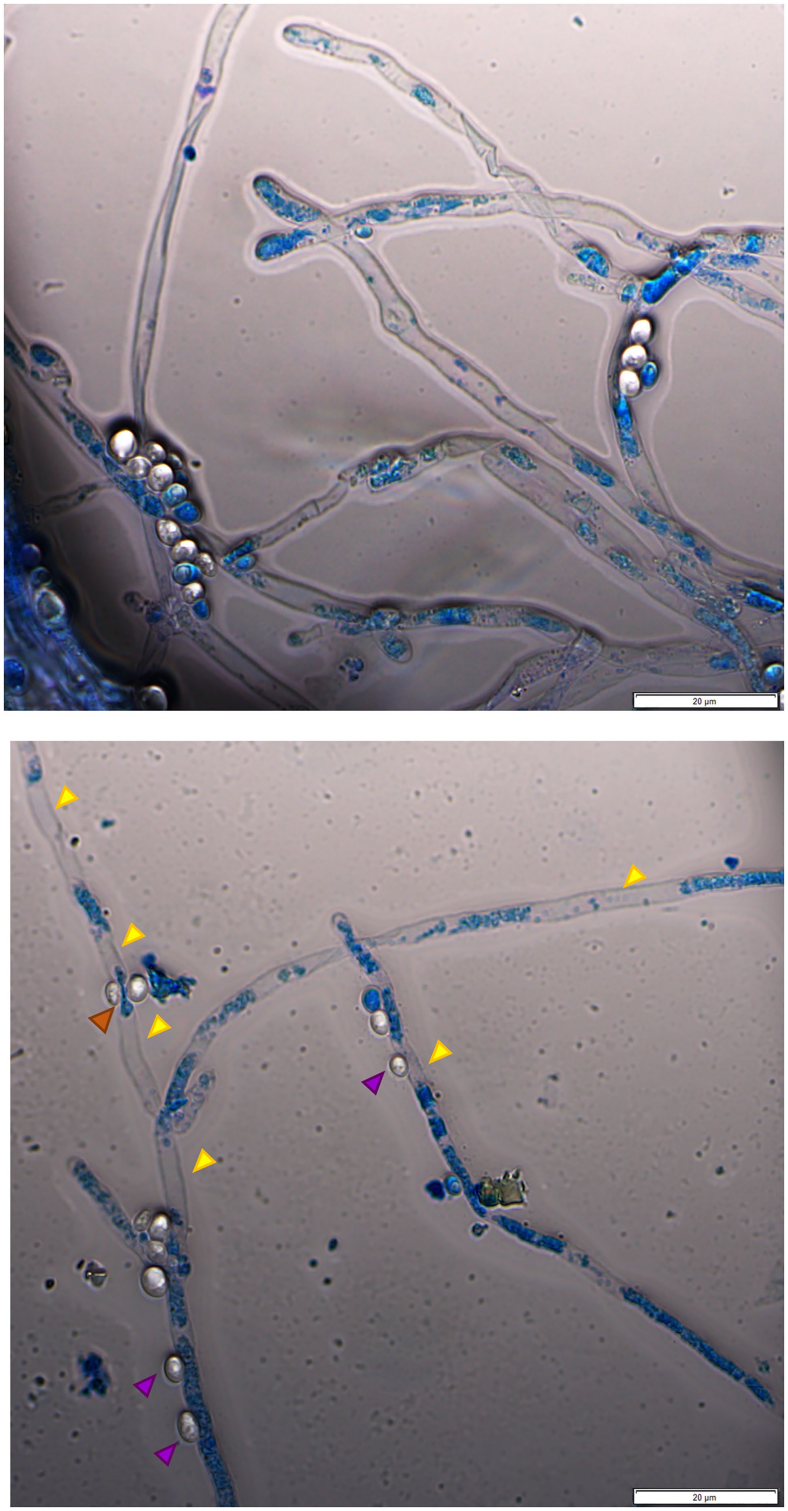
Fluorescence microscopy image of *W. anomalus* #1105 and *M. perniciosa* CBS 442.80 co-culture stained with MB. The yeasts rather group while adhering to the hyphae (**upper panel**). The hyphae are partially drained of their cellular content (**upper panel and lower panel**, yellow arrows). **Lower panel**: yeast cells appear to push the hypha inwards (orange arrow); yeasts may cause a constriction of the hypha (purple arrow).

Assays in solid media show fungal growth inhibition. The filamentous fungi were fully active and alive in the opposite direction to that where the yeast was standing (Figure 1A), and the MB dye in the agar did not show that there was death of the fungal culture in those circumstances. To verify whether the antagonism observed in liquid medium implicates the death of the mycelium or just the inhibition of mycelium development, co-cultured cells for 10days were stained with MB and PI. All the yeasts/*M. perniciosa* strains combinations classified as level 1 in Table 3 showed the mycelium partially stained with either dye, while all the combinations classified as level 2 presented an almost fully stained mycelium with both MB and PI (exemplified in Figure 3). In all cases, the yeasts appear to adhere to the hyphae, but in level 2 combinations, the fluorescence microscopy images showed isolated or clustered adhering yeast cells causing an inwards deformation and a constriction of the hypha (exemplified in Figure 4, orange and purple arrows, respectively). In those cases, it was also possible to see that the hyphae were drained of their cellular content becoming empty and flattened (exemplified in Figure 4, yellow arrows). This suggests that in liquid media, the physical contact between the two organisms might be part of the mechanism that causes fungal death, which was accompanied or concomitant with cell draining, which might not necessarily occur through lysis since the walls of the hyphae appear to remain intact.

### Are Yeasts Truly Adhering to the *M. perniciosa* Hyphae and Draining Them?

**Figure 5:**
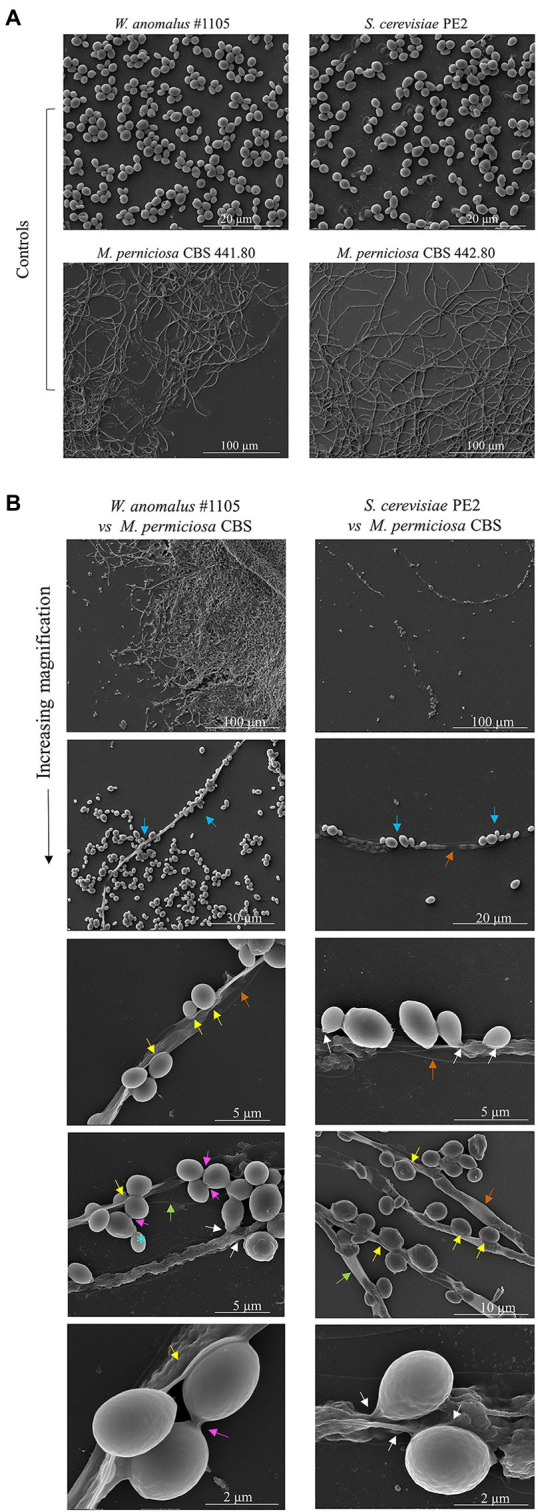
SEM micrographs of *W. anomalus* #1105 *vs* CBS 441.80 and *S. cerevisiae* PE2 *vs* CBS 442.80 co-cultured cells. **(A)** Controls images of yeasts and filamentous fungi growing separately in MEA at 30°C. **(B)** Increasing magnification micrographs show: the yeasts rather group while adhering to the hyphae (blue arrows); hyphae are drained (orange arrows); yeasts push into a hypha (yellow arrows); yeasts fuse with hyphae (white arrows). Bottom micrograph showing high magnification of *W. anomalus* #1105 shows that the yeast cells are ligated to each other (pink arrow), differently than from an incomplete bud separation (blue*).

To further investigate the morphological characteristics of this yeast/*M. perniciosa* interaction, chosen combinations were observed by SEM. Specific attention was paid to the yeast-hyphae contact zone. Micrographs of *W. anomalus* #1105 and *S. cerevisiae* PE2 co-cultured with *M. perniciosa* CBS 441.8 and 442.8, respectively, are shown in increasing magnifications in Figure 5. Images show that the yeast cells adhere to the hyphae more often in groups of two or more cells than individually, as previously observed by fluorescence microscopy with MB staining (Figure 4).

**Figure 6:**
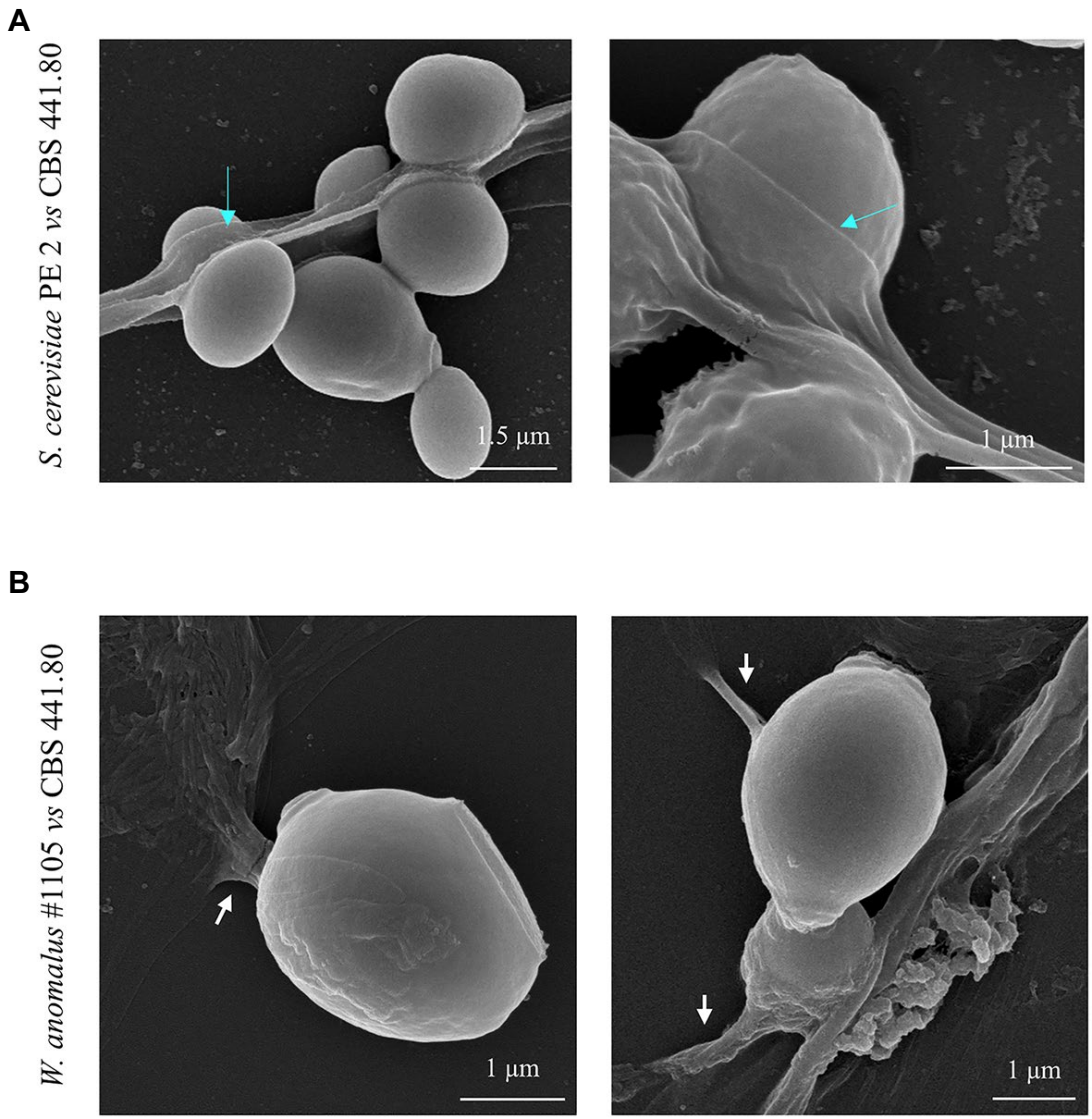
SEM micrographs of the yeast/filamentous fungal strains combinations **(A)** *S. cerevisiae* PE2 vs. *M. perniciosa* CBS 441.80 and **(B)** *W. anomalus* #1105 vs. *M. perniciosa* CBS 442.80, showing in detail the occurrence of the *veil* covering the yeast cells (blue arrows), and the apparent tube-like structures ligating yeasts to hyphal contents (white arrows).

**Figure 7:**
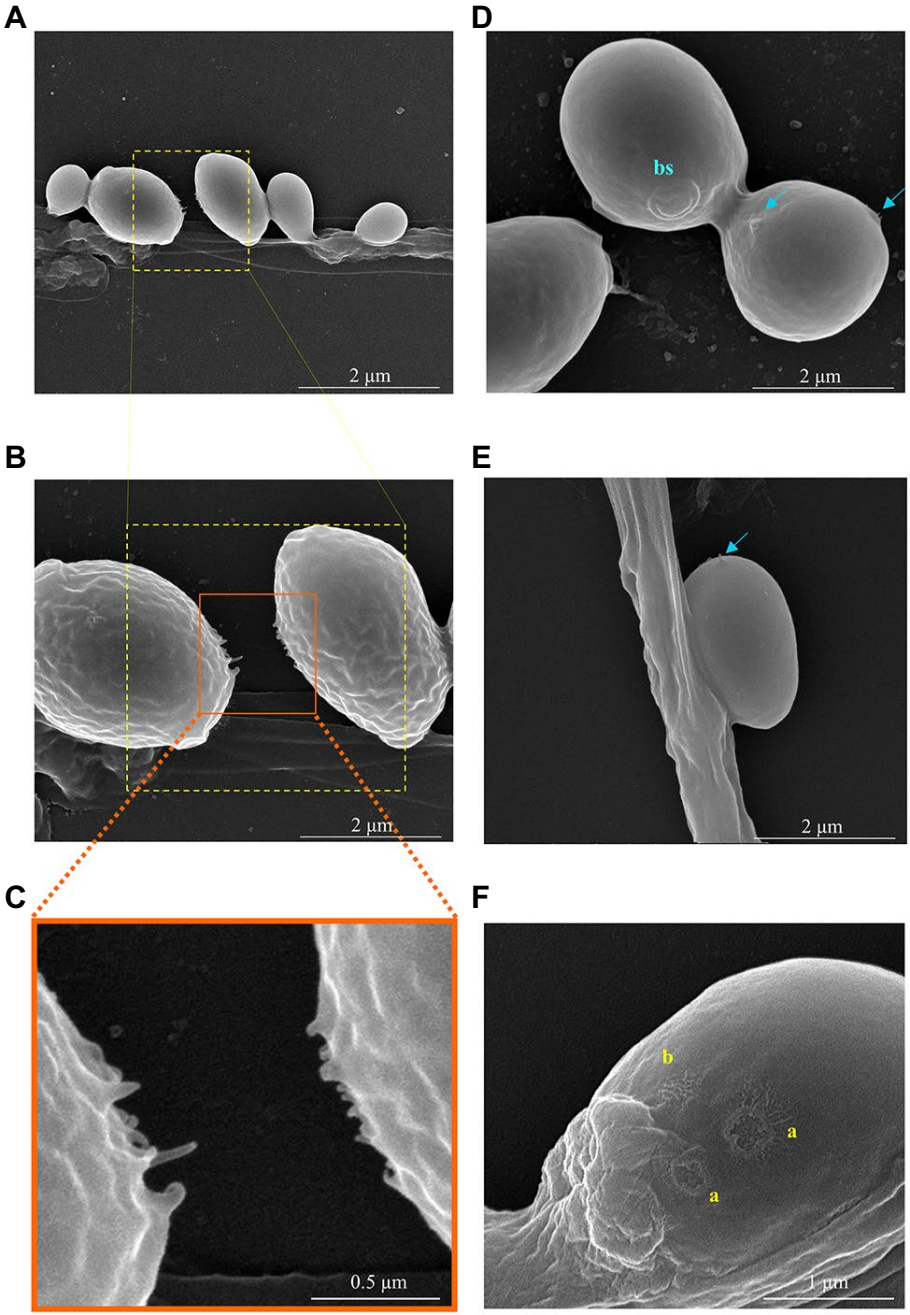
SEM micrographs showing the details of the interaction between two *W. anomalus* #1105 yeast cells during the antagonistic action against *M. perniciosa* CBS 442.80. **(A-C)** Increasing magnification of the fimbriae-like structures connecting two yeast cells. **(D,E)** Blue arrows indicate forming protrusions; bs stands for bud scar having app. 700nm Ø. An internal granulated structure is visible, each grain with app. 30–40nm Ø. **(F)** Yellow letters indicate the scars of previous sections connecting yeasts, which present two different shapes (a) open/recent scar with 200–300nm Ø and (b) probably a closed scar.

The SEM analysis yet revealed additional structures in association with the yeast-hypha interaction, presented in detail in the micrographs of Figures 6, 7, formed between the cells of *W. anomalus* #1105 and the hyphae of CBS 441.80, but not between *S. cerevisiae* PE2 with CBS 442.8. The first is a veil-like form (Figure 6A, blue arrows) smoothly covering the yeast cell adhering to the hypha. The second, more prominent structure observed, consisted of a tube-like connection between the cells of the yeast and the hyphae (Figure 6B, white arrows). To the best of our knowledge, interspecies physical communication is extremely rare, although it occurs between predacious yeasts and their prey (Lachance and Pang, 1997; Lachance et al., 2012). The connection tubes now observed could be related to the mutual recognition by the two microorganisms or be associated with the possible invasion of the *M. perniciosa* cells by the antagonistic yeasts. A third unprecedented structure is shown in Figure 7. As mentioned above, yeasts rather adhere to the hyphae in groups than alone (Figure 5). Their grouping appears to be more than just backing to each other. Fimbriae-like connections between yeast cells are formed, around a roughly circular area with approximately 0.2–0.3μm Ø (Figures 7A–C, top-to-bottom increasing magnification). Their location in the cell surface appears to be random (Figures 7D,E, blue arrows). Importantly, they do not appear to be permanent. Cells eventually separate, leaving a small scar (Figure 7F, yellow letters), smaller than a bud scar and with a different morphology. It is roundish, with a small rim around the edges of a shallow cavity which contains numerous protrusions with app. 20–30nm Ø. This structure might be transient and reabsorbed into the cell smooth surface after detachment, as suggested by the difference between the two morphologies a and b in Figure 7F. Interestingly, Mamvura et al. (2017) published a SEM micrograph of a mixed biofilm formed inside a brewery equipment, in which yeast cells formed a large number of fibrils connecting each other while supposedly feeding on bacteria. No specific structure is visible connecting bacteria and yeasts though.

## Discussion

*W. anomalus* #1105 (previously *Pichia anomala* or *Hansenula anomala)* and *S. cerevisiae* #1112 from spontaneous fermentations, used to produce *cachaça* (da Conceição et al., 2015), and the *S. cerevisiae* strain PE2, widely used in Brazil in the industrial production of bioethanol (Lopes et al., 2016), were revealed in this study as good candidates for the utilization as biocides in the management of WBD. This conclusion was based on that (i) they efficiently antagonize *M. perniciosa*, originating from cacao plantations in Brazil and other South American countries afflicted with WBD, inhibiting fungal growth in solid media, and killing the fungus in liquid media, and (ii) they maintained their antagonistic ability at three different temperatures, under starvation, at different culture stages, or growing old.

This study aimed at identifying yeasts that perform as strong biocides of the *M. perniciosa*, so that their utilization in the field can be envisaged. Indeed, yeasts were found active against the filamentous fungus *M. perniciosa* that causes the WBD of cacao fruits and trees. Yeasts, particularly *S. cerevisiae* strains, are often employed in the postharvest protection of fruits and other food products against fungal spoilage (Nally et al., 2015; Parafati et al., 2015; Pretscher et al., 2018), which raises the possibility that they might be also used for the preharvest control of phytopathogens (Lopes et al., 2015). Only one such formulation exists commercially registered, Romeo®, which contains *S. cerevisiae* cell wall components that induce fungal resistance in the plant, and is recommended for the control of mildew and botrytis on grapevine and vegetables (Freimoser et al., 2019). Yeasts are found in soil, sediments, and water, in plant surfaces and fruits, and in insect guts. Yeasts are also colonizers of plants as endophytes, which contributes to the raising interest in these microbes for preharvest, phytopathology purposes (Joubert and Doty, 2018). Their ubiquitous presence as part of the natural microbial ecosystems might be an advantage. Their biodiversity can therefore be explored to find a suitable biocidal strain that is not harmful to each crop’s biome. Nevertheless, prospecting the free survival of yeasts in the environment, where they will suffer harsh and/or sudden changes in temperature, humidity, and possibly starvation, the focus should be on yeasts that are naturally more resilient and better known. These were the premises underlying this work’s choice of yeast strains.

*M. perniciosa* was previously shown to be inhibited *in vitro* by two yeast isolates (Cabral et al., 2009). The disease was most severe in Brazil (Lisboa et al., 2020), which has a well-established tradition of yeast-based biotechnology (Barbosa et al., 2016; Lopes et al., 2016). Therefore, a group of yeast strains originating from those processes was chosen to prospect for antagonism against *M. perniciosa*, bearing in mind they share with the fungal strains a broad subequatorial geographical origin and climate. Moreover, since these are yeast strains used by the industry, their commercialization and acceptance should be facilitated. Besides geography, other criteria were used to choose the yeast strains. Those included their predominance during a fermentative process (da Conceição et al., 2015; Barbosa et al., 2016; Lopes et al., 2016; Monteiro et al., 2018), which is a good indication of their resilience, mandatory for successful introduction in nature where they have to survive sharp environmental changes and large periods of nutrients deprivation and drought. This predominance has often been attributed to a strong Killer ability against other yeasts and bacterial contaminants (reviewed by Klassen et al., 2017). Both *W. anomalus* and *S. cerevisiae* were previously described in the literature as fungal antagonists, secreting siderophores or other chemicals, volatiles, or diffusible, some of which reduce spore germination or decrease the length of germ tube, or hydrolytic enzymes that destroy the fungal cell wall, or competing for nutrients, space, and biofilm formation (De Ingeniis et al., 2009; Nally et al., 2015; Parafati et al., 2015; Oro et al., 2018; Pretscher et al., 2018). In particular, their Killer strains were described to act upon fungal phytopathogens (Suzzi et al., 1995; Walker et al., 1995; Cabral et al., 2009; Rosa-Magri et al., 2011; Platania et al., 2012; Lima et al., 2013; Liu et al., 2013). Therefore, yeasts were additionally chosen according to the strength of their Killer phenotype (da Conceição et al., 2015), although their mode of action against *M. perniciosa* may be the result of several processes occurring simultaneously (Walker, 2011; Pretscher et al., 2018; Freimoser et al., 2019).

This study showed substantial differences between the antagonism responses obtained in solid and liquid media. This may derive from several factors. Liquid medium propitiates the growth of the yeast population, faster than that of the fungal mycelium, favoring the competition for nutrients, which was reported as one of the main modes of yeast-phytopathogenic filamentous fungus antagonism (Andrews et al., 1994; Saravanakumar et al., 2008; Zhang et al., 2010; Spadaro and Droby, 2016). Accordingly, the inhibition of mycelium formation does not imply death, being often reversible due to fungal cells retaining their viability (Spadaro and Droby, 2016). Otherwise, yeasts remain metabolically active for a longer period in liquid medium, which allows more homogenous distribution of the yeast cells and their easier contact with the hyphae which may trigger the production and secretion of compounds with antifungal properties. In this study, the dual staining with MB and PI of *M. perniciosa* mycelium in liquid co-cultures with yeasts evidenced the death of the fungus, suggesting the possible disruption of the hyphae plasma membrane. Fluorescence microscopy analysis further revealed that (i) the yeasts adhere to the fungal hyphae, more often in groups of two or more yeast cells than alone, (ii) the point of contact between a yeast cell and a hypha is deformed, forming a concavity, and (iii) the hyphae appear to be drained of their contents. This was further confirmed using SEM to analyze *W. anomalus* #1105 and *S. cerevisiae* PE2 antagonism, which clearly confirmed these observations. These features closely resemble those of predacious yeasts upon other yeasts (Lachance and Pang, 1997; Lachance et al., 2012). There are only a few yeasts described to behave as predators, all of which belonging to a common clade which contains a majority of *Saccharomycopsis*, a few *Candida* and one *S. cerevisiae* species (Lachance et al., 2012). A few cases are described in the literature in which the yeast prey is a fungal phytopathogen, namely *Botrytis cinerea, Podosphaera xanthii* or *Penicillium* sp. *(*reviewed by Freimoser et al., 2019). Yeast predation of other yeasts or fungal hyphae acts upon their prey through the development of a penetration peg/haustorium (Lachance and Pang, 1997). SEM analysis does not enable to observe this kind of structure, but it allowed to see that the yeasts eventually fuse with the hyphae, which is emptied and flattened without evidence of chaotic cell disruption and lysis. These results suggest that the yeasts might literally predate the fungus, feeding on their intracellular components. Such a behavior needs not be independent of the secretion of antifungal compounds or hydrolytic enzymes, which may act synergistically, facilitating predation (Nally et al., 2015; Junker et al., 2018). In that case, those subsidiary mechanisms should not be able *per se* to replace efficiently the predation-associated yeast-hyphae adhesion and invasion (Pretscher et al., 2018). Accordingly, in the present study, the supernatants of yeast cultures and of yeast-filamentous fungus co-cultures were unable to inhibit mycelium development or to kill the fungus.

Previously, *W. anomalus* was described aggregating in large clumps of cells around the hyphae of *Botryodiploidia theobromae* (Hashem and Alamri, 2009) and *Colletotrichum gloeosporioides* (Lima et al., 2013; Zepeda-Giraud et al., 2016). Although other yeasts were mentioned to be also able to adhere to hyphae in a similar fashion, the attachment affinity was highest for *W. anomalus* (Hashem and Alamri, 2009). In the case of *C. gloeosporioides,* the yeast cells were reported to fuse with the hyphae (Zepeda-Giraud et al., 2016), which were drained (Lima et al., 2013), identically to what was observed in the present study. Importantly, *W. anomalus* and *M. guilliermondii* were previously shown to cause an accentuated deformation of the hyphae while adhering as the one observed in the present study, described as a concavity or a pit (Chan and Tian, 2005; Hashem and Alamri, 2009; Lima et al., 2013; Zepeda-Giraud et al., 2016). This hyphae deformation has been attributed to the action of hydrolytic enzymes, which degrade the cell wall by making perforations, causing the general weakening of the cell (Walker, 2011; Lima et al., 2013; Zepeda-Giraud et al., 2016). This is though not the single mode of action of *W. anomalus*, since this yeast is able to secrete Killer toxins or VOCs besides enzymes, as well as of acidifying the medium and compete for nutrients, and is generally more resilient to a number of stress factors than the target fungi (Walker, 2011). In sum, several authors suggest that the yeasts use several mechanisms to attack fungi, including mycoparasitism (Lachance et al., 2012; Dukare et al., 2018; Freimoser et al., 2019).

The adhesion of *W. anomalus* was also associated with the secretion of mucilage (Hashem and Alamri, 2009; Zepeda-Giraud et al., 2016) as this study’s SEM observations also suggest. Yeasts connecting physically through some kinds of fibrils are characteristic of biofilms, which formation demands the secretion of a viscous mucilage that acts as an extracellular matrix (ECM), providing adhesion, support, and commanding the diffusion of molecules and cell differentiation (e.g., Vogel, 2018; Karygianni et al., 2020). The veil observed to partially cover the yeasts attached to the hyphae could correspond to such a mucilage, secreted to improve adhesion. Because it is observed in most of the hyphal-adhering yeast cells, it is improbable that it corresponds to an artifact from the fixation process. Accordingly, biofilm-derived yeast cells are more efficient to antagonize filamentous fungi than their planktonic counterparts as a consequence of the secretion of ECM (Freimoser et al., 2019).

The present SEM analysis yet revealed other important features associated with yeast-hypha interaction. The cells of either *W. anomalus* or *S. cerevisiae* PE2 tend to group while adhering to the hyphae, that is, during the killing of the fungus, but only those of *W. anomalus* displayed this fimbriae-like physical bonds between each other. In view of the different size of the scars observed in the cell surface, cells could be eventually detaching from each other and subsequently closing the correspondent *wound*. This suggests that their predatory action over the filamentous fungus might demand for a collective strategy and that their alliance could be transitory. Detached cells show the existence of fimbriae-like structures bordering a roundish attachment area filled with protuberances which have an estimated diameter compatible with that of microtubules. In bacteria, fimbriae and *pili* are known for being involved in the adherence to inert surfaces or living tissues, but importantly they are also involved in cell-cell communication (reviewed by Berne et al., 2018). In the case of yeasts, information in this regard is scarce. Connection fibrils between two yeast cells were shown to form in biofilms of *Candida* sp. (Furlaneto et al., 2012) or *S. cerevisiae* (Varon and Choder, 2000; Mamvura et al., 2017; Freimoser et al., 2019) in which case they were suggested to have 180±50nm Ø, similar to the ones observed in the present study. In some *Candida* species, they were attributed to have a role in the development of colonies (Vargas et al., 2004; Furlaneto et al., 2012). In *S. cerevisiae*, they were associated with starvation or aging (Varon and Choder, 2000). Varon and Choder (2000) and Mamvura et al. (2017) suggested that the connections between the yeasts within a colony or a biofilm promote a high degree of collective organization for a common good, which lasts only while yeasts are experiencing long periods of stress like starvation or dehydration. If they are re-fed, or when they are young and living in abundant nutrients, that type of high order organization does not occur. Concurrently, studies on yeasts social behavior demonstrated that yeasts inside a biofilm are capable of cooperation (reviewed by Crespi, 2001 and Wloch-Salamon, 2014). Some of the studies that revealed cooperative behavior between yeasts were done in a population of yeasts thriving in a sucrose-alone medium and were based on that the secretion of invertase is guaranteed by only a fraction of the fermenting population. Curiously, the sugarcane juice-fermenting communities from which the yeasts strains used in the present work were isolated are all sucrose-alone cultures. Whether long-term adaptation to sucrose preconditions yeasts behavior toward a more cooperative behavior, to the best of our knowledge, has never been addressed. In opposition, the release of Killer toxins has been classified as “interference competition,” selfish behavior (Wloch-Salamon et al., 2008). If two or more yeasts cooperate for effective predation of a fungus, that would mean a more complex level of social interaction than those described so far in association with yeasts populations, possibly more in the line of cooperative attacks done by *Myxobacteria* (Dworkin, 1996; Crespi, 2001).

SEM also revealed the formation of tube-like structures apparently connecting yeast to hypha. There is no description available in the literature of the formation of specific structures connecting the cytoplasm of two cells of different species as it was observed in our study between the yeast and the hypha. These structures could be similar to animal cells actin polymerization-driven protrusions like filopodia (Faix and Rottner, 2006), to fungal pegs that invade plant tissues (Li et al., 2016), or even to the penetration peg of predacious yeasts on their prey (Lachance and Pang, 1997). In any case, it would promote invasion of the hyphae and feeding. Whether this is the case, and whether they are structurally and functionally different from the fimbria described connecting yeast cells (Varon and Choder, 2000), remains to be clarified in the future. It also remains to be seen whether yeast-yeast and/or yeast-hypha connections involve the protrusion of microtubules/actin filaments as it appears. Bacteria and higher eukaryotic cells, both, are able to connect through intercellular membrane tunneling nanotubes (TNT; Onfelt et al., 2006; Pande et al., 2015; Matkó and Tóth, 2021). Bacteria and archaebacteria form a network of these filaments which allow a whole community of cells to cross-feed (Pande et al., 2015) and share signaling molecules and other compounds (Bassler and Losick, 2006; Pande et al., 2015) or even vesicles (Onfelt et al., 2006; Delage and Zurzolo, 2013). Yeasts might share with bacteria the same collective survival strategy.

## Conclusion

This study assessed a set of yeast strains originating from sugarcane juice-based fermentative processes for their ability to antagonize and kill strains of *M. perniciosa*, the filamentous fungus causing cacao WBD. Three yeast strains were identified as efficient and resilient biocides, belonging to the *S. cerevisiae* and the *W. anomalus* species, which are both considered safe for human manipulation by the regulatory agencies FDA and EFSA. This study also presents a new in depth on the mode by which these yeasts antagonize the fungus, in particular *W. anomalus* which behavior is compatible with that of a necrotrophic mycoparasite. Importantly, the microscopy assessment revealed that most possibly this yeast acts in the realm of a collective strategy, that involves the formation of physical structures connecting yeast cells with each other and with the hyphae, suggesting a cooperative predation and cross-feeding. Although the work ultimately focused on two yeast strains, *W. anomalus* LBMC1105 and *S. cerevisiae* PE2, all the remaining strains used in this study were able to antagonize *M. perniciosa* to some extent. The other way around, although the work focused on two *M. perniciosa* strains from Brazil (CBS 441.80 and 442.80), the chosen three yeasts were also able to efficiently antagonize the four remaining South American *M. perniciosa* strains. This highlights the robustness of the proposed use of yeasts as biocides to manage cacao WBD. Considering that sugarcane is fermented to produce spirits as a generalized cultural habit throughout Central and South American countries or to produce ethanol to feed the energy matrix of Brazil, it is plausible to consider that fermentations might supply wild yeasts able to locally counteract cacao’s WBD. Using local yeast strains to manage the disease would thus contribute to the economic sustainability of the smaller cacao producers from those regions that have less or no access to agrochemicals or other pest management strategies, while raising expectations as to their environmental low impact. This study is in that regard the initial step toward the formulation of a new eco-friendly and effective alternative for controlling WBD, consisting of the application of live yeast suspensions, without the need for the purification of a specific antifungal compound.

## Data Availability Statement

The raw data supporting the conclusions of this article will be made available by the authors, without undue reservation.

## Author Contributions

PF: investigation, methodology, validation, writing – original draft, and writing – review and editing. RB: supervision and resources. FC: supervision and writing – review and editing. CL: conceptualization, supervision, writing – original draft, and writing – review and editing. All authors contributed to the article and approved the submitted version.

## Funding

This study was supported by the strategic program UID/BIA/04050/2020 funded by national funds through the FCT I.P., and by the ERDF through the COMPETE2020 – Programa Operacional Competitividade e Internacionalização (POCI) and the project AgriFood XXI (NORTE-01-0145-FEDER-000041). This study was also supported by the Norte Portugal Regional Operational Programme (NORTE 2020). PF was a PhD student of the Doctoral Program in Applied and Environmental Microbiology (DP_AEM; FCT grant no. PD/BD/113814/2015).

## Conflict of Interest

The authors declare that the research was conducted in the absence of any commercial or financial relationships that could be construed as a potential conflict of interest.

## Publisher’s Note

All claims expressed in this article are solely those of the authors and do not necessarily represent those of their affiliated organizations, or those of the publisher, the editors and the reviewers. Any product that may be evaluated in this article, or claim that may be made by its manufacturer, is not guaranteed or endorsed by the publisher.

## Footnotes

^1^ www.wi.knaw.nl
