# Supplementary figures and images for: Moniliophthora perniciosa, the Causal Agent of Cacao Witches’ Broom Disease Is Killed in vitro by Saccharomyces cerevisiae and Wickerhamomyces anomalus Yeasts
Source: Front Microbiol. 2021 Sep 22;12:706675. doi: 10.3389/fmicb.2021.706675 (PMC8493218; doi:10.3389/fmicb.2021.706675)

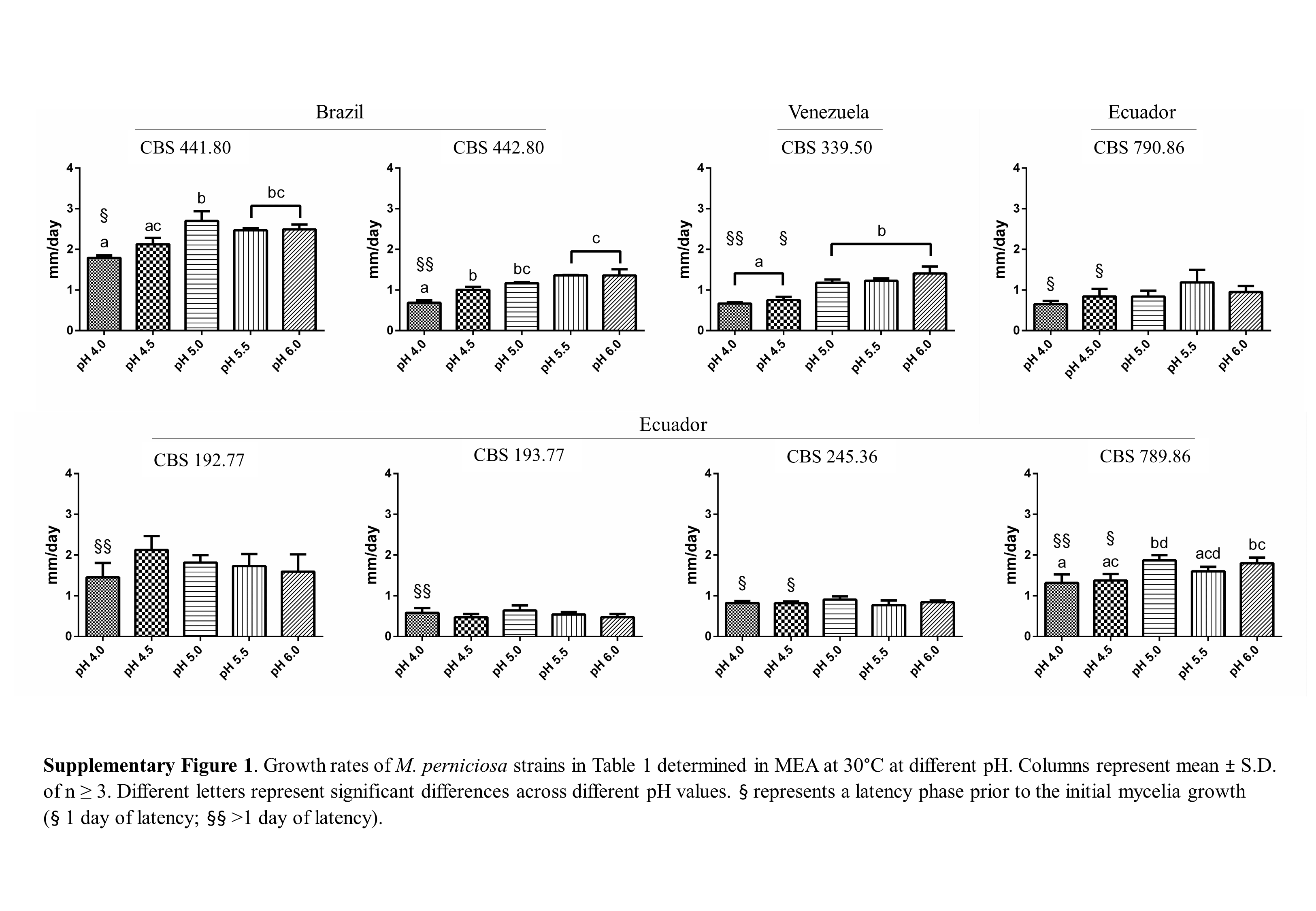

Supplement: Supplementary file 1 [file Image_1.TIF]
